# Observational skills assessment score: reliability in measuring amount and quality of use of the affected hand in unilateral cerebral palsy

**DOI:** 10.1186/1471-2377-13-152

**Published:** 2013-10-21

**Authors:** Lucianne Speth, Yvonne Janssen-Potten, Pieter Leffers, Eugene Rameckers, Anke Defesche, Richard Geers, Rob Smeets, Hans Vles

**Affiliations:** 1 Adelante, Paediatric Rehabilitation, Onderstestraat 29, 6301 KA, Valkenburg, the Netherlands; 2 Adelante, Centre of Expertise in Rehabilitation and Audiology, Hoensbroek, the Netherlands; 3 Maastricht University, Research School CAPHRI, Department of Rehabilitation Medicine, Maastricht, the Netherlands; 4 Maastricht University, Research School CAPHRI, Department of Epidemiology, Maastricht, the Netherlands; 5 Maastricht University Medical Centre, Department of Rehabilitation Medicine, Maastricht, the Netherlands; 6 Maastricht University Medical Centre, Department of Neurology, Maastricht, the Netherlands; 7 Maastricht University, research school GROW, Department of Neurology, Maastricht, the Netherlands

**Keywords:** Cerebral palsy, Upper limb, Bimanual performance, Outcome assessment, Reliability

## Abstract

**Background:**

The Observational Skills Assessment Score (OSAS) measures amount and quality of use of the affected hand in children with unilateral Cerebral Palsy (CP) in bimanual activities and could therefore be a valuable addition to existing assessment tools. The OSAS consists of tasks that are age appropriate and require use of the affected hand.

**Methods:**

To measure the agreement and reliability of the OSAS a convenience sample of two groups of 16 children with unilateral spastic CP (2.5-6 and 12–16 years old), performed age specific bimanual tasks in 2 measurement sessions. Three experienced raters took part in testing and 8 in scoring. Intra class correlation (ICC) values for intra- and inter-rater reliability, and the mean and standard deviation of the differences between measurements were calculated. For test-retest reliability beside ICC scores, Smallest Detectable Differences (SDDs) were calculated in 16 older and 10 younger children.

**Results:**

Generally, there seems to be good agreement between repeated measurements of the OSAS, as indicated by the small SDDs on most scales for quality of movement, compared to the range of their scales. This indicates potentially good sensitivity to change if used for patient evaluation purposes. The exceptions were the ‘quality of reach’ score for all tasks, and all quality scores for the stacking blocks task for the young children. As used in the present study, the OSAS has good discriminative capacity within patient populations as indicated by the high ICCs for most quality scores. Measuring the amount of use does not seem to be useful for either discrimination or evaluation.

**Conclusion:**

In general, the OSAS seems to be a reliable tool for assessing the quality of use of the affected hand in bimanual activities in younger and older children with unilateral CP. Some modifications may improve its usefulness and efficiency.

## Background

Children with clinically apparent unilateral Cerebral Palsy (CP) have specific hand function problems. If they use their affected hand, it is always as an assisting hand. Even with only minor impairment of their affected hand, they often do not use it to its full potential in bimanual tasks. This is called developmental disregard [1-4]. Therefore, evaluation of hand function over time or after treatment should focus on the actual use of the affected hand in bimanual activities of daily life, i.e. bimanual performance, as well as on the ability to use the affected hand to its maximal potential in bimanual tasks performed in a standardized environment, which is called capacity [5,6].

Several assessment tools have been developed for children with unilateral CP. Gilmore et al. [7] reviewed the psychometric properties and clinical utility of several upper limb measures at the International Classification of Functioning, Disability and Health (ICF) activity level [8]. They concluded that the Melbourne Assessment of Unilateral Upper Limb Function (MUUL) [9] is superior for measuring unilateral capacity, while the Assisting Hand Assessment (AHA) [10] and ABILHAND-Kids questionnaire [11] have the best psychometric properties for measuring bimanual performance. Only the AHA is considered to be sufficiently responsive [12].

In 2005 we studied the effect of botulinum toxin A (BoNT-A) injections on upper limb functional skills in children with unilateral CP [13]. We used the MUUL as primary outcome measure, because, at that time, it was the best available tool at the ICF activity level. No effect of BoNT-A could be demonstrated. In hindsight this is not surprising because the MUUL contains many items relating to target accuracy, and these are unlikely to be influenced by BoNT-A. Furthermore, it measures one hand at a time and contains tasks that are usually not done by the assisting hand. In 2003, the AHA was specifically developed to assess the effective use of the assisting hand in bimanual performance. Use of the affected hand is stimulated but not obligated in the AHA. We therefore felt the need for an instrument that measures the capacity of the affected hand in bimanual activities and so developed the Observational Skills Assessment Score (OSAS) using basic ideas of the Video Observation Aarts and Aarts (VOAA) [14]. Whereas the MUUL measures unilateral capacity and the AHA measures actual use in bimanual performance, the OSAS measures both the amount and the quality of use (capacity) of the affected hand in tasks in which both hands need to be used. Task performance, especially in young children, can be influenced by visual spatial insight, praxis and cognitive aspects. The OSAS’ tasks were therefore chosen to be appropriate for the children’s ages and their intellectual abilities to prevent these factors from affecting task performance. They involve many repetitions of actions so that quality of use can be assessed reliably. Task execution is filmed, allowing blind assessment. The amount and quality of use of the affected hand are scored every second in order to make the OSAS more sensitive to subtle differences in these features than the AHA. In the AHA, the performance that is observed most frequently during the play session, or sometimes the best performance, is scored.

The OSAS is still under development. It is intended for use in clinical practice to support choice of treatment and for treatment evaluation as well as for research purposes. Because, as a first step in its evaluation, it is important to know its ability to give the same results in repeated measurements, we assessed intra-rater, inter-rater and test-retest agreement and reliability using the Guidelines for Reporting Reliability and Agreement Studies (GRRAS) [15].

### Development and description of OSAS

An expert team of three occupational therapists, three physiotherapists and one rehabilitation physician developed several age-appropriate, standardized bimanual motor tasks for children of 2.5 to 6 and 7 to 16 years old. These tasks cannot be performed by the child without repetitively using the affected hand. In the younger age group, the tasks are building with ‘Pop-Onz’ (Fisher Price®), threading beads and stacking blocks. For the older children, the tasks are small and large screw and nut construction, and buttering and cutting bread. The child’s performance is videotaped, allowing blind assessment.

### OSAS administration protocol

The OSAS manual provides an exact description per task of how the child should be positioned at the table, table height, position of the materials needed, and what instructions should be given to the child. This manual is available from the corresponding author; an example is given in Additional file 1. Two synchronized cameras are used. Simultaneous frontal and cranial views make assessment of the use of the affected hand easier, because the positions of wrist, thumb and fingers are easier to see. The correct positioning of the cameras is also described in the manual. Administration of the three tasks takes about 45 minutes.

### OSAS scoring protocol and data processing

The quality of use of the affected hand is scored on an ordinal scale. Four domains of use were defined: reach, grasp (position thumb and fingers, and position wrist), hold (position thumb and fingers, and position wrist), and release. Each domain has 3 to 5 quality score levels ranging from poor to good quality. The same expert group that designed the tasks also formulated the quality criteria. These criteria are described in detail in Additional file 2.

**Figure 1:**
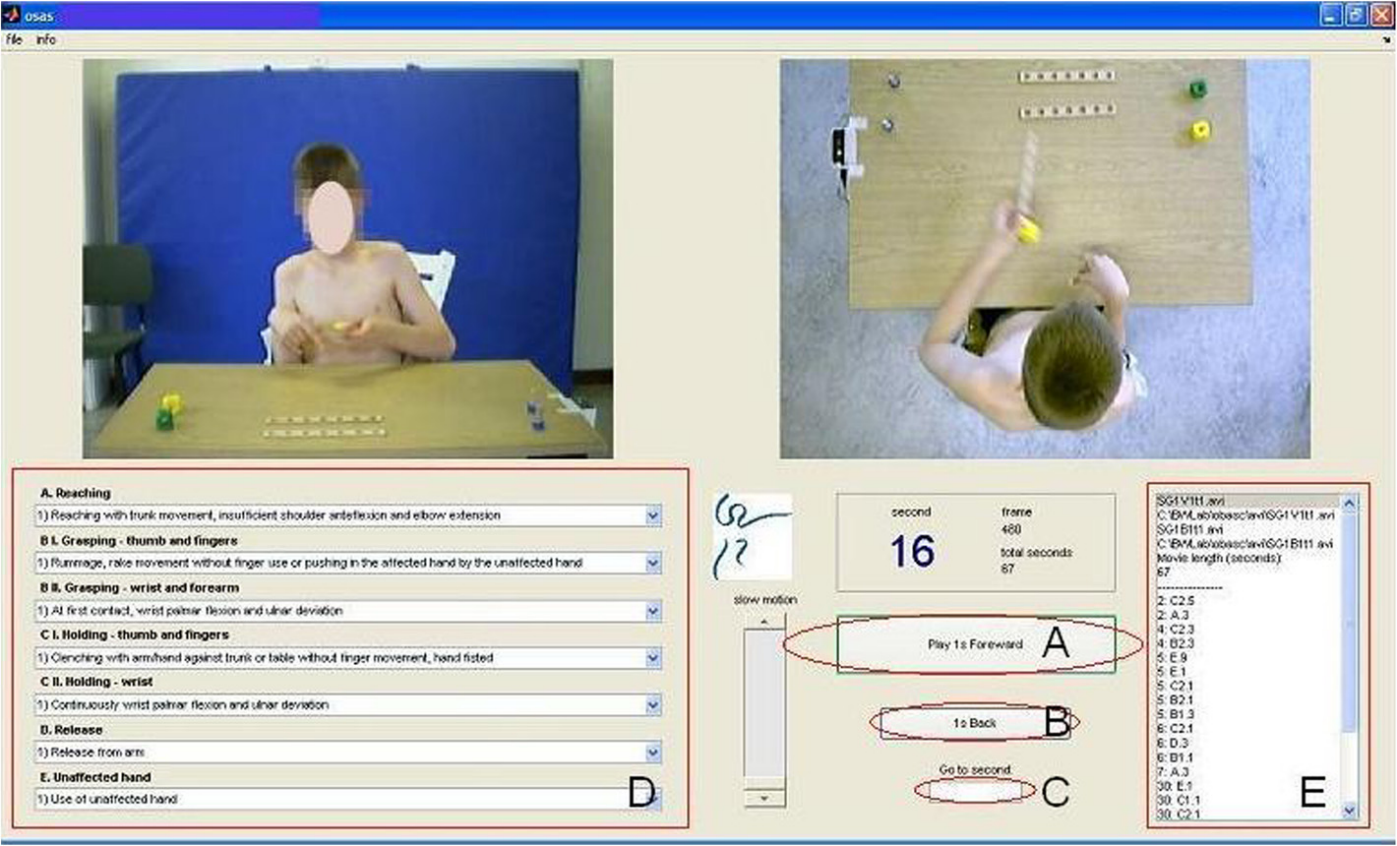
OSAS video recordings with quality criteria for scoring as a popup menu (given with the parents’ written permission).

The video recordings are analyzed with a user-dedicated software program (Figure 1) based on MATLAB (MathWorks inc). The frontal and cranial views are displayed next to each other. In the same window the quality criteria for scoring are shown as a pop-up menu. The video recordings are forwarded second-by-second. Every second both the quality of use of the affected hand and the use of the non-affected hand are scored according to the previously mentioned quality criteria. Scoring the video recording of one task takes 20 minutes for an experienced rater. To prevent scoring from taking too long, each task is limited to a maximum of 2.5 minutes. All participants were able to complete the tasks within this time. At least one of the quality criteria of the affected hand has to be scored every second unless the affected hand was not used. In that case, only the use of unaffected hand is scored. The amount of use of both hands during the task is expressed as a proportion of the total time needed to complete the task. The mean quality score of each domain has to be calculated separately for each task; a higher mean score corresponds to better quality of hand function.

## Methods

### Participants and study design

A convenience sample of 16 children aged 12 to 16 years (older age group) and 16 children aged 2.5 to 6 years (younger age group) with unilateral spastic CP performed the tasks. The older children participated in a constrained induced therapy program and the younger children in a BoNT-A and/or specific therapy effect study. Children and parents gave their written informed consent to use data for research purposes. The study in which the children participated had medical ethics approval of the METC Atrium-Orbis-Zuyd (06-p-33) and the Dutch CCMO (ref: NL12005.096.06). Due to the study design and the therapy program, children with very severe hand impairments classified as Manual Ability Classification Score (MACS) IV or V [16], who were not able to use their hand, were not enrolled in this reliability study. We used their baseline measures before any intervention took place. Three experienced therapists performed these measurements. In the older age group 5 children had a MACS I, 9 children a II and 2 had a MACS III score. In the younger age group 4 had MACS I, 8 MACS II, and 4 MACS III. All children were intellectually able to perform these tasks.

### Raters

**Figure 2:**
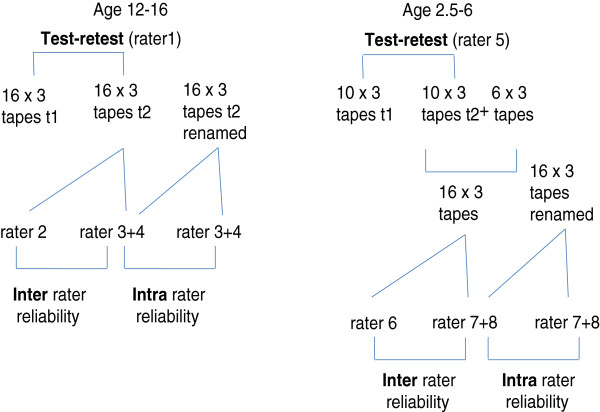
Reliability assessments. Rater 3 + 4 and 7 + 8 each scored half of the 2 times 16 (× 3 tasks) tapes.

The three tasks for the 16 older children were performed twice during 2 sessions with 6 weeks in between in which no intervention took place. Considering their age, we did not expect them to change within this period. In the younger age group, because of the protocol of the study in which they were enrolled, the same was done with 10 children with 2 weeks between measurements. The videotapes of these task performances were scored by the same rater later for the test-retest reliability assessment. For 16 children from both the older and younger age groups, task performance of the second test session was scored by two raters to assess inter-rater reliability. The videotape of this second session task was renamed to allow twice blind scoring by the same raters with at least two weeks in between to determine intra-rater reliability (Figure 2). The raters were physiotherapists or occupational therapists trained by an occupational therapist who is a co-developer of the OSAS. This training consisted of scoring videotapes of these tasks performed by other CP children not involved in this study. This was first done in a group session and later practiced as individual home work exercises. There were 4 raters for both age groups; all raters scored the videotapes independently.

### Statistics

After data processing with MATLAB, the proportion of time that both hands were used during tasks (i.e. the percentage of use of both hands), and mean scores of the quality of use of the affected hand on the different domains (reach, grasp fingers, grasp wrist, hold fingers, hold wrist and release) were determined. The mean differences of the two measurements and their standard deviations (SD) were computed. Intra Class Correlation (ICC) values (95% confidence interval) with a two-way random-effects analysis of variance model (absolute agreement type) of these mean scores were computed to determine the intra-rater, the inter-rater and test-retest reliability of both amount and quality of use [17]. In addition we calculated the Standard Error of Measurement (SEM) and the Smallest Detectable Difference (SDD) as measures of agreement [18]. The SDD was calculated as 1.96 × √2 × SEM [17] and represents the threshold that must be overcome to ensure that a change is real.

## Results

### Means and ranges

**Figure 3:**
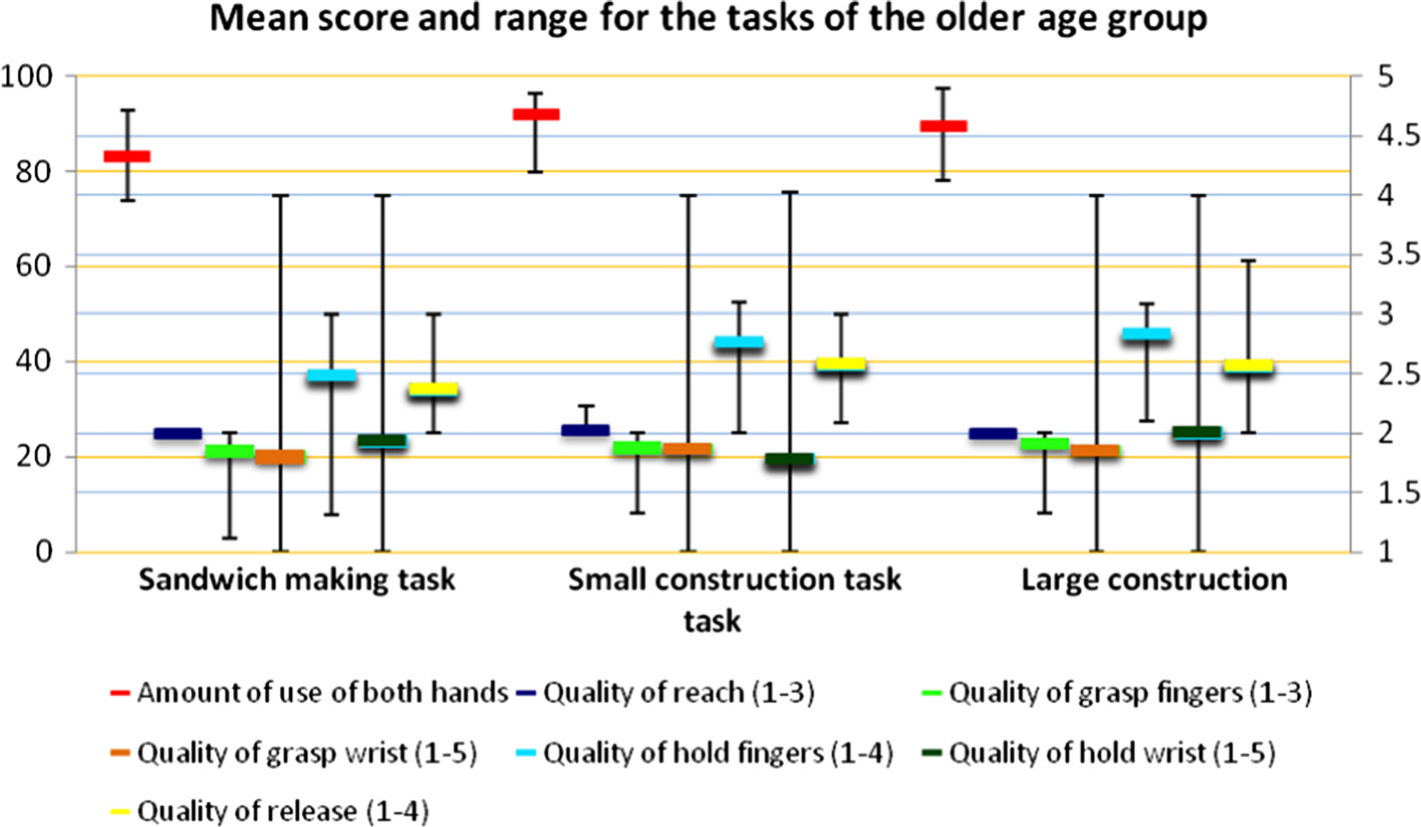
**Mean score and range for the tasks of the older age group.** Left Y-axis is amount of use (%, orange lines), right Y-axis is quality of use (blue lines).

**Figure 4:**
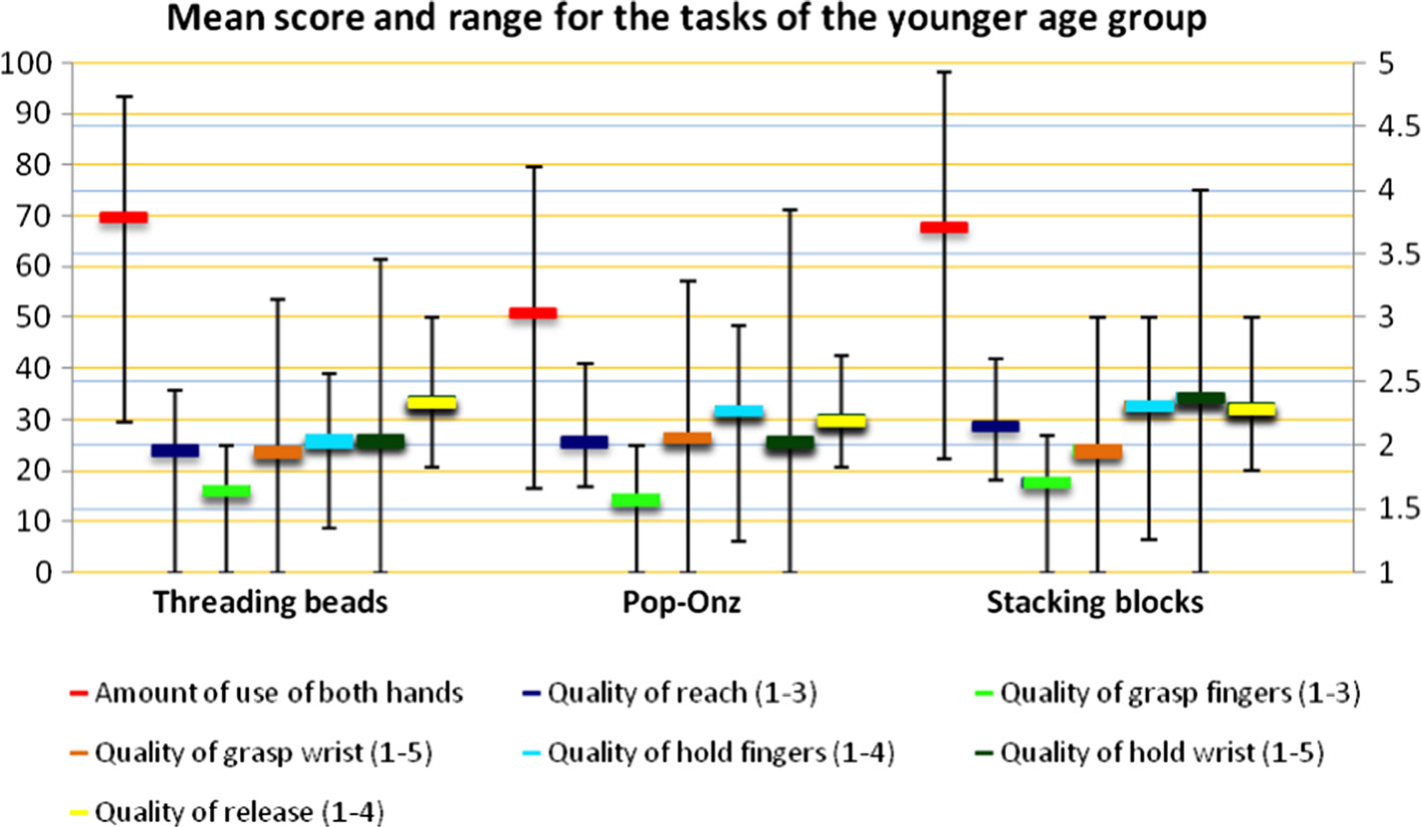
**Mean score and range for the tasks of the younger age group.** Left Y-axis is amount of use (%, orange lines), right Y-axis is quality of use (blue lines).

The means and ranges for the amount and quality of use scores are shown in Figure 3 for the older age group and in Figure 4 for the younger age group. The amount of use of both hands by the older children was high, with little variation between children (Figure 3), especially in the construction tasks. With the younger children the amount of use of both hands was clearly lower and showed more variation.

For quality of reach, the score was almost always 2 in the older age group; for the younger children there was some variation. In the older age group the quality of grasp wrist score had a larger range and the quality of hold fingers score was clearly higher, whereas the quality of hold wrist score was slightly lower and had a larger range compared to the younger children (Figure 4).

### Reliability data

#### Older age group

The intra-rater, inter-rater and test-retest reliabilities expressed as ICC values (95% confidence interval) of the percentage of use and mean quality scores of the three fine motor tasks of the 16 older children are shown in Tables 1, 2 and 3, respectively. Table 3 also shows the SDD of the percentage of use of both hands and the mean scores of the quality domains.

**Table 1:** Intra-rater reliability of amount of use and quality of use in the older age group

	***Amount of use***	***Quality of use affected hand***					
	**Use of both hands (%)**	**Quality of reach (1–3)**	**Quality of grasp fingers (1–3)**	**Quality of grasp wrist (1–5)**	**Quality of hold fingers (1–4)**	**Quality of hold wrist (1–5)**	**Quality of release (1–4)**
**Making a sandwich**							
ICC	0.960	NE	0.977	0.972	0.900	0.987	0.960
95% CI	0.890-0.986	1 ch. diff.	0.936-0.992	0.922-0.990	0.741-0.964	0.963-0.995	0.890-0.986
Mean diff.	−0.12	0.00	0.01	0.00	−0.03	−0.02	−0.02
SD difference	1.64	0.01	0.06	0.27	0.19	0.18	0.09
**Construction task small**							
ICC	0.857	0.899	0.976	0.992	0.878	0.999	0.844
95% CI	0.635-0.948	0.737-0.964	0.933-0.992	0.978-0.997	0.693-0.955	0.996-1.0	0.615-0.942
Mean diff.	0.87	0.00	−0.00	−0.02	−0.06	−0.02	−0.06
SD difference	2.02	0.04	0.05	0.14	0.16	0.05	0.16
**Construction task large**							
ICC	0.953	NE	0.086	0.970	0.865	0.984	0.749
95% CI	0.873-0.983	1 ch. diff.	−0.390-0.539	0.920-0.989	0.655-0.951	0.956-0.994	0.429-0.903
Mean diff.	−0.30	0.04	−0.06	−0.07	0.07	−0.16	−0.07
SD difference	1.60	0.09	0.19	0.27	0.16	0.21	0.25

ICC = intra class correlation; CI = confidence interval, SD = standard deviation, NE = not executable; 1 ch. diff. = the rater scored only 1 child differently.

**Table 2:** Inter-rater reliability of amount of use and quality of use in the older age group

	***Amount of use***	***Quality of use affected hand***					
	**Use of both hands (%)**	**Quality of reach (1–3)**	**Quality of grasp fingers (1–3)**	**Quality of grasp wrist (1–5)**	**Quality of hold fingers (1–4)**	**Quality of hold wrist (1–5)**	**Quality of release (1–4)**
**Making a sandwich**							
ICC	0.877	NE	0.019	0.945	0.657	0.913	0.841
95% CI	0.691-0.955	6. ch. diff.	−0.498-0.507	0.852-0.980	0.270-0.864	0.772-0.968	0.600-0.941
Mean diff.	−0.94	0.05	0.06	−0.06	0.16	0.07	−0.01
SD difference	2.87	0.08	0.42	0.40	0.36	0.44	0.18
**Construction task small**							
ICC	0.785	0.124	0.400	0.955	0.815	0.964	0.722
95% CI	0.472-0.920	−0.297-0.546	−0.112-0.741	0.879-0.984	0.543-0.931	0.902-0.987	0.372-0.893
Mean diff.	1.13	0.11	0.04	−0.11	−0.01	−0.02	0.05
SD difference	2.27	0.25	0.27	0.37	0.25	0.29	0.28
**Construction task large**							
ICC	0.826	NE	0.108	0.944	0.485	0.967	0.687
95% CI	0.567-0.936	5 ch. diff.	−0.358-0.550	0.846-0.980	−0.017-0.787	0.908-0.988	0.319-0.877
Mean diff.	−0.10	0.18	−0.08	−0.16	0.01	−0.03	−0.08
SD difference	3.11	0.33	0.22	0.40	0.30	0.32	0.31

ICC = intra class correlation; CI = confidence interval; SD = standard deviation; NE = not executable; n ch. diff. = the rater scored n children differently.

**Table 3:** Test-retest reliability of amount of use and quality of use in the older age group

	***Amount of use***	***Quality of use affected hand***					
	**Use of both hands (%)**	**Quality of reach (1–3)**	**Quality of grasp fingers (1–3)**	**Quality of grasp wrist (1–5)**	**Quality of hold fingers (1–4)**	**Quality of hold wrist (1–5)**	**Quality of release (1–4)**
**Making a sandwich**							
ICC	0.446	NE	0.845	0.965	0.804	0.914	0.830
95% CI	−0.032-0.762	4 ch. diff.	0.607-0.943	0.904-0.988	0.515-0.928	0.744-0.971	0.558-0.938
Mean diff.	−1.50	−0.02	−0.07	0.02	−0.14	−0.21	−0.09
SD difference	5.75	0.05	0.16	0.29	0.29	0.39	0.17
SDD	11.30	0.10	0.34	0.55	0.61	0.85	0.36
**Construction task small**							
ICC	0.038	0.064	0.640	0.979	0.808	0.932	0.789
95% CI	−0.436-0.515	−0.358-0.514	0.211-0.862	0.941-0.993	0.531-0.931	0.816-0.977	0.475-0.924
Mean diff.	−2.40	−0.04	0.03	0.05	0.08	0.12	−0.04
SD difference	7.18	0.08	0.18	0.24	0.22	0.41	0.25
SDD	14.37	0.17	0.35	0.47	0.44	0.81	0.47
**Construction task large**							
ICC	0.441	NE	0.074	0.936	0.744	0.969	0.692
95% CI	−0.008-0.755	2 ch. diff.	−0.464-0.549	0.832-0.977	0.401-0.903	0.914-0.989	0.305-0.881
Mean diff.	−2.97	−0.00	0.01	0.12	0.01	0.04	0.00
SD difference	7.00	0.12	0.20	0.41	0.19	0.30	0.30
SDD	14.50	0.23	0.39	0.81	0.37	0.58	0.58

ICC = intra class correlation; CI = confidence interval; SD = standard deviation; NE = not executable; n ch. diff. = the rater scored n children differently; SEM = standard error of measurement; SDD = smallest detectable difference.

In the older age group the proportion of time that both hands were used (*amount of use*, second column) showed high intra- and inter-rater reliability ICC values, varying from 0.79 to 0.96. The test-retest ICC values showed low agreement (0.04, 0.44, and 0.45). Correspondingly, mean differences and their SDs were also larger than in intra- and inter-rater reliability measurements. The SDD of the amount of use of both hands in these older children varied from 11.3% (making a sandwich) to 14.5% (construction task large). These numbers indicated high variation in relation to the mean percentages of use of both hands (83% – 92%, see Figure 3).

The ICC values expressing intra-rater reliability of *quality of use* for all domains (Table 1, column 3 to 8) for the three tasks of the 16 older children varied from 0.75 to 0.99, indicating good reliability. Only the ICC of the quality of grasp finger score of the large construction task was low: 0.09. The ICCs for ‘the quality of reach’ of the sandwich making and the large construction tasks could not be calculated because too many mean scores were identical. We therefore reported the mean difference between the two measurements and its standard deviation. A difference in the mean of ‘quality of reach’ (Table 1) only emerged in one child.

In the older age group, inter-rater reliability of the quality of use showed good ICC scores for all measurements except for ‘quality of reach’ and ‘quality of grasp fingers’. The SDs of the difference in these measurements were higher than in the test-retest measurements (Table 2).

For test-retest reliability, high ICCs were found in most tasks. The ‘quality of reach’ score of the small construction task and the ‘quality of grasp fingers’ score of the large construction task had low ICC values. The ICCs for the ‘quality of reach’ of the sandwich making and large construction task could not be calculated, because there was too little variation. The SDD of the quality scores varied from 0.10 to 0.85.

#### Younger age group

Tables 4, 5 and 6 present intra and inter-rater (16 children) and test-retest reliability (10 children) ICC values of the means for the three tasks of the younger children. Table 6 also shows the SDD.

**Table 4:** Intra-rater reliability of amount of use and quality of use in the younger age group

	***Amount of use***	***Quality of use affected hand***					
	**Use of both hands (%)**	**Quality of reach (1–3)**	**Quality of grasp fingers (1–3)**	**Quality of grasp wrist (1–5)**	**Quality of hold fingers (1–4)**	**Quality of hold wrist (1–5)**	**Quality of release (1–4)**
**Threading beads**							
ICC	0.989	0.587	0.842	0.694	0.933	0.716	0.784
95% CI	0.969-0.996	0.134-0.0.835	0.615-0.941	0.311-0.882	0.823-0.976	0.366-0.890	0.479-0.919
Mean diff.	−0.21	−0.02	−0.05	0.04	0.03	−0.14	−0.01
SD difference	2.75	0.30	0.16	0.60	0.10	0.62	0.24
**Pop-Onz**							
ICC	0.969	0.728	0.106	0.807	0.933	0.865	0.585
95% CI	0.914-0.989	0.365-0.900	−0.432-0.570	0.527-0.928	0.825-0.976	0.665-0.970	0.156-0.831
Mean diff.	−0.22	0.04	−0.04	0.00	0.07	−0.12	−0.07
SD difference	4.81	0.20	0.40	0.52	0.22	0.42	0.28
**Stacking blocks**							
ICC	0.888	0.311	0.496	0.818	0.791	0.775	0.664
95% CI	0.717-0.959	−0.195-0.689	0.018-0.790	0.559-0.932	0.493-0.922	0.471-0.915	0.189-0.864
Mean diff.	2.20	0.13	0.07	−0.21	0.01	−0.11	−0.19
SD difference	9.02	0.53	0.34	0.55	0.35	0.66	0.29

ICC = intra class correlation; CI = confidence interval; SD = standard deviation.

**Table 5:** Inter-rater reliability of amount of use and quality of use in the younger age group

	***Amount of use***	***Quality of use affected hand***					
	**Use of both hands (%)**	**Quality of reach (1–3)**	**Quality of grasp fingers (1–3)**	**Quality of grasp wrist (1–5)**	**Quality of hold fingers (1–4)**	**Quality of hold wrist (1–5)**	**Quality of release (1–4)**
**Threading beads**							
ICC	0.932	0.078	0.619	0.624	0.530	0.527	0.684
95% CI	0.796-0.977	−0.194-0.442	0.213-0.846	0.196-0.851	0.097-0.802	0.048-0.806	0.308-0.876
Mean diff.	−3.0	0.66	-.0.12	−0.07	−0.14	−0.09	−0.13
SD difference	5.72	0.77	0.26	0.67	0.34	0.82	0.28
**Pop-Onz**							
ICC	0.943	0.173	0.117	0.774	0.823	0.676	0.293
95% CI	0.847-0.980	−0.211-0.575	−0.264-0.527	0.465-0.915	0.0.561-0.934	0.281-0.874	−0.153-0.666
Mean diff.	−2.07	−0.37	−0.20	−0.06	0.02	0.06	−0.19
SD difference	6.10	0.58	0.36	0.54	0.37	0.64	0.43
**Stacking blocks**							
ICC	0.950	0.295	0.709	0.826	0.557	0.740	0.228
95% CI	0.864-0.982	−0.109-0.656	0.341-0.888	0.577-0.935	0.122-0.817	0.404-0.901	−0.128-0.596
Mean diff.	−0.86	0.31	0.03	−0.11	0.17	0.13	−0.53
SD difference	5.76	0.37	0.28	0.53	0.58	0.77	0.56

ICC = intra class correlation; CI = confidence interval, SD = standard deviation.

**Table 6:** Test-retest reliability of amount of use and quality of use in the younger age group

	***Amount of use***	***Quality of use affected hand***					
	**Use of both hands (%)**	**Quality of reach (1–3)**	**Quality of grasp fingers (1–3)**	**Quality of grasp wrist (1–5)**	**Quality of hold fingers (1–4)**	**Quality of hold wrist (1–5)**	**Quality of release (1–4)**
**Threading beads**							
ICC	0.720	0.118	0.770	0.906	0.770	0.899	0.787
95% CI	0.248-0.921	−0.598-0.686	0.294-0.938	0.666-0.976	0.347-0.936	0.665-0.974	0.337-0.943
Mean diff.	−4.90	−0.08	0.003	0.02	−0.09	−0.10	0.01
SD difference	12.36	0.59	0.20	0.35	0.25	0.37	0.24
SDD	24.90	1.11	0.37	0.65	0.50	0.71	0.44
**Pop-Onz**							
ICC	0.689	0.693	0.604	0.934	0.597	0.847	0.379
95% CI	0.157-0.912	0.127-0.921	0.062-0.881	0.771-0.983	−0.051-0.884	0.488-0.960	−0.361-0.805
Mean diff.	3.75	0.14	0.11	0.07	0.02	0.01	−0.01
SD difference	16.11	0.20	0.24	0.28	0.54	0.47	0.29
SDD	30.82	0.43	0.49	0.55	1.0	0.87	0.53
**Stacking blocks**							
ICC	0.775	0.401	0.511	0.665	0.554	0.592	0.116
95% CI	0.304-0.939	−0.283-0.809	−0.153-0.853	0.072-0.906	−0.054-0.866	−0.015-0.880	−0.626-0.688
Mean diff.	−0.08	−0.07	−0.06	−0.03	−0.13	−0.18	0.005
SD difference	12.19	0.30	0.31	0.82	0.46	0.78	0.48
SDD	22.65	0.57	0.59	1.53	0.89	1.49	0.89

ICC = intra class correlation; CI = confidence interval, SD = standard deviation; SEM = standard error of measurement; SDD = smallest detectable difference.

In the younger age group the *amount of use* of both hands during the tasks showed high ICC values for intra-, inter-rater, and test-retest reliability, varying from 0.69 to 0.99. The SDDs of the amount of use of all tasks with these children were clearly larger (22.7% to 30.8%) than with the older children, and were highest in the Pop-Onz task. In this task especially, the mean amount of use of both hands was low (Figure 4) and had a wide range. The differences between the first and second measurement varied from 1.3 to 35.5. The larger range and greater variation in differences explain these higher SDD scores.

The intra-rater reliability of *quality of use* of the three tasks for the 16 younger children showed high ICC values varying from 0.59 to 0.93. The exceptions were the ‘quality of reach’ during the stacking blocks task and ‘quality of grasp fingers’ in the Pop-Onz and stacking blocks tasks.

The inter-rater reliability in this age group showed good ICC’s for most of the measurements except for ‘quality of reach’ of all the tasks, ‘quality of grasp fingers’ of the Pop-Onz task, and ‘quality of release’ in the Pop-Onz and stacking blocks tasks.

For test-retest reliability of quality of use for the younger children good ICC scores were found for most tasks but ‘quality of reach and release’ of several tasks indicated less agreement. The SDD of the quality of use domains varied from 0.43 and 0.44 (‘quality of reach’ Pop-Onz and ‘quality of release’ threading beads) to 1.49 and 1.53 (‘quality of hold and grasp wrist’ stacking blocks).

## Discussion and conclusions

We designed the OSAS to measure both the amount and quality of use of the affected hand in tasks in which repetitive bimanual use is demanded. It was developed primarily to measure treatment effect in research and clinical practice. In the present study, as a first evaluation, intra-rater, inter-rater and test retest reliability and agreement were determined using ICC, standard deviation of measurement differences and SDD [17,18].

Because the ICC provides an index that relates to distinguishing patients within a specific group its importance for clinical evaluation of change is limited. However, based on the generally high ICC values in most tasks, it can be concluded that the OSAS has good discriminative capacity in patient groups resembling the study population. For the older children, an exception in this general pattern is the amount of use of both hands for which the variation is small in all tasks, which explains the low ICCs in test-retest reliability. The mean differences between the measurements for inter- and intra-rater reliability are generally small compared to the width of their scales, which indicates good agreement of measurements.

The largest SDD for the amount of use of both hands was 14.5% in the older age group and 30.8% for the younger children. As the SDD uses the same units as the original measurement, its interpretation for clinical use is straightforward. The high amount of use in the older children, in combination with large SDDs leads to a ceiling effect, rendering this measurement non-useful for follow-up. Because the OSAS demands the use of both hands to perform the tasks, this is not surprising. In the younger age group, in which movement patterns are not yet very stable, there was less use of both hands, more variation and large SDDs. This means that amount of use of the OSAS in younger children is not suited for evaluating individual changes but may still be used to compare groups in scientific research.

The ‘quality of reach’ has very low variation, which leads to low reliability in both age groups. The lack of variation may be explained by the fact that children with unilateral CP tend not to reach with their affected hand. If they do, it is only for a second and almost always with the same pattern. For the future it would be better to score the fact that the affected hand reached without an attached quality criterion.

The ICC of quality of use is good to excellent for ‘grasp wrist’, ‘hold fingers’, and ‘hold wrist’ in all children and all tasks. This is also the case for ‘quality of release’ in all tasks in the older age group and the threading beads task in the younger children. They do not yet show very consistent release patterns, which is especially obvious during the Pop-Onz and stacking blocks tasks. The ICC of ‘quality of grasp fingers’ is not good in the Pop-Onz task of the younger, and in the small and large construction tasks (only inter-rater reliability) of the older children. This means that better coaching of the observers in this criterion will be needed. Generally, the reliability for the older age group is better than for the younger children. This may be explained by the fact that older children show more consistent movement patterns. The SDDs of the quality items are generally small, but clearly larger in the stacking blocks task of the younger children. The largest SDDs are found in the ‘quality of grasp’ and ‘hold wrist’ mean score in the stacking blocks task. Therefore this task is not very useful to measure change. Apart from ‘reach’ the SDDs of the quality criteria for the OSAS tasks are low compared to the width of their scales which makes these criteria potentially useful for assessing change in patients.

The OSAS seems to be a useful addition to existing assessments of bimanual functioning for children with unilateral hand function problems, such as the AHA. The AHA measures actual spontaneous use of the affected hand in bimanual performance. With the OSAS, the amount and quality of use of the affected hand can be measured in a precise way, as a measure of capacity. The tasks are designed to force the child to use the affected hand repeatedly, are appropriate for the age group and do not interfere with visual spatial or praxis problems. In contrast to the MUUL, the OSAS measures the affected hand as an assisting hand in bimanual functioning. The simplicity and the short duration of the tasks make the OSAS easier to administer with young children. A disadvantage is that scoring takes longer, 20 minutes per task.

In the present study, 32 children between the age of 2.5 and 16 years were included in the intra- and inter-rater reliability analyses and 26 children in the test-retest reliability analysis. This number is limited. Moreover, children aged 7–11 years old were not included in the present study. However, part of the OSAS was developed for children aged 7–16 years old. Reliability data from this age group will need to be collected.

More agreement data is needed, with adapted scoring of the reach item. Only the frequency of reaching with the affected hand during the task can be scored. The stacking blocks task, which proved to be unreliable, might be removed. Precise coaching of observers is needed, especially for the assessment of ‘quality of grasp fingers’. The next evaluation step is to measure concurrent validity. In the children aged twelve years or younger this is possible with the AHA. Because the AHA is not available for the older age group yet, the Jebsen test [19], which measures speed of movement of the affected hand, could be used instead. Concurrent validity will also be determined with the achievement of treatment goals assessed by the Goal Attainment Scaling (GAS) [20] and performance scores of the COPM [21].

In conclusion, the OSAS appears to be a reliable assessment tool, with good agreement between repeated measurements, for measuring the quality of use of the affected assisting hand in forced bimanual task execution in CP children. Some modifications as mentioned above, may improve agreement, reliability and ease of scoring. More agreement and reliability data should be gathered, and the responsiveness of the scores also needs to be tested.

## Competing interests

The authors declare that they have no competing interests.

## Authors’ contributions

LS is first corresponding author and participated in drafting the manuscript. She made main contributions to design, acquisition, analysis and interpretation of data. YJ and PL have been involved in drafting the manuscript, analysis and interpretation of data and critically revising it. ER and AD were involved in data acquisition and development of the OSAS. RG designed the user-dedicated software program of the OSAS. RS and HV critically revised the intellectual content of the article. All authors read and approved the final manuscript.

## Pre-publication history

The pre-publication history for this paper can be accessed here:

http://www.biomedcentral.com/1471-2377/13/152/prepub

## Supplementary Material

Additional file 1 **Building using construction material.** Age 7- 16 years. Click here for file

Additional file 2 OSAS quality criteria. Click here for file
