# Supplementary material for: Observational skills assessment score: reliability in measuring amount and quality of use of the affected hand in unilateral cerebral palsy
Source: BMC Neurol. 2013 Oct 21;13:152. doi: 10.1186/1471-2377-13-152 (PMC3853931; doi:10.1186/1471-2377-13-152)
Supplement: Additional file 1 — Building using construction material. Age 7- 16 years. [file 1471-2377-13-152-S1.pdf]

Building using construction material
Age 7- 16 years

Setting
Quiet room
Rectangular table with a hard tabletop that is adjustable in height
Table and chair adjusted to the correct height of the child
Webcam 1 stands in front of the table and provides a mid frontal view, approximately on shoulder height of the child: head, trunk, arms, hands and the entire working space come on screen
Webcam 2 is mounted on the ceiling above the table for a cranial view, both hands are visible.

This set-up is valid for a child with unilateral pareses on the right.

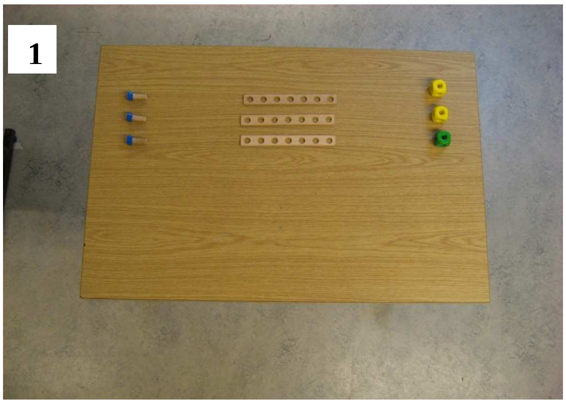

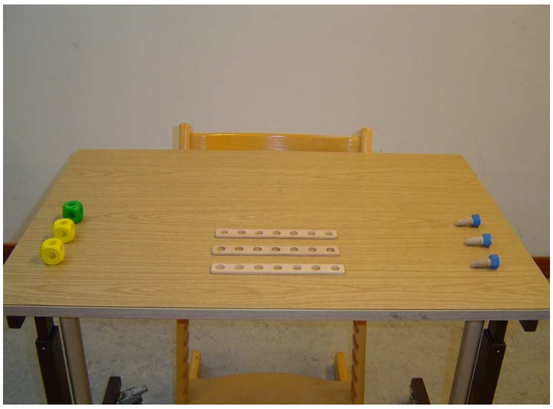

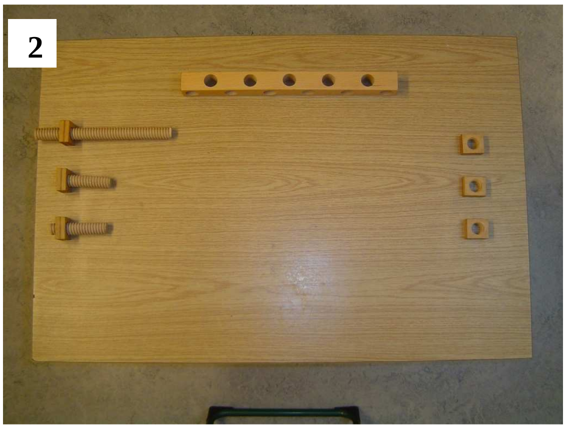

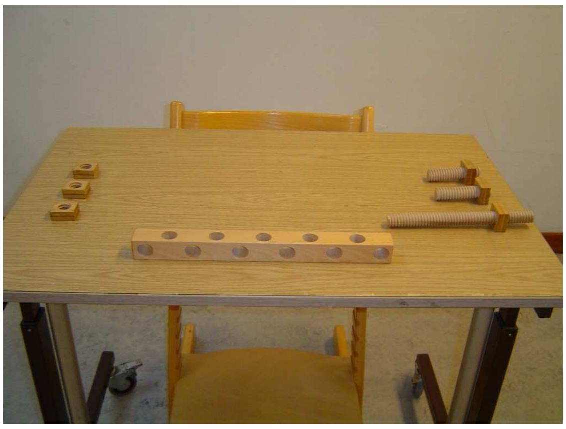

Cranial view **Frontal view**

Material:
- Session 1 small material : 3 screws, 3 little blocks, 3 slats (brand Baufix, base set)
- Session 2 big material: 3 sticks (2 short and one long stick) with screw thread and female screws, 3 female screws and one little beam 30 cm long.

Starting position
The child sits behind the table with both arms on the table. The chair and table are adjusted to the right height of the child. Both feet are on the floor or on a footrest. The child's upper body is undressed or is dressed in an undershirt.
Female screws and blocks are ready on both sides of the table.

Session 1:
- 3 slats lay in front of the child, in horizontal direction and within reaching distance of the child
- 3 coloured blocks lay on the table, on the affected side of the child
- 3 screws lay on the table, on het non-affected side of the child

Session 2:
- The little beam lays in front of the child, in horizontal direction and within reaching distance of the child
- 3 female screws lay on the table, on the affected side of the child, within reaching distance of the child
- 3 sticks with screw thread and female screws lay on the non-affected side of the child, within reaching distance of the child (the 2 short sticks closest to the child and the long stick furthest away from the child, see picture 2)

Instruction to the child
The assessor tells the child he/she is going to build with construction material.
Make sure the aim of het task is clear to the child and that the child understands how the task has to be performed.

Session 1:
"You may build using the construction material. Attach 1 coloured block to the slat using a screw. Attach the block to the first (or last) whole. Attach 1 block to each slat. Attention: 2 wholes of the blocks do not have screw thread!
You may start when I say 'go'".
The assessor counts down: 3-2-1-go; at three the video starts recording, at 'go' the child starts performing the task.

When the child has finished the task, he/she puts the last slat with a block attached down on the table and the video recording stops.

Max time: 2.5 minutes.

Session 2:
“You may build again. Attach a screw to the little beam using a female screw. Attach the other two screws to the little beam in the same manner (all female screws must be on the same side of the beam). You may start when I say ‘go’”.
The Assessor counts down: 3-2-1-go; at three the video starts recording, at ‘go’ the child starts performing the task.

When the child has attached all three crews to the little beam, he/she puts the beam down on the table and the video recording stops.

Max time: 2.5 minutes

Video instruction in case of problems:

- In case the child does not know how to attach the screws, first give verbal instruction (e.g. put the screw into the block, turn the other way, choose a different whole in het block etc.), then indicate and finally demonstrate the child how the task is being done.
- In case the child has forgotten what has to be attached to what, repeat the instruction, or indicate.
- In case the child uses only one hand: do not respond to that.

Maximal time: 5 minutes
Max time per task: 2 ½ minutes.
Max time both tasks total: 5 minutes.

Scoring:
According to the quality criteria.
