# Supplementary material for: Observational skills assessment score: reliability in measuring amount and quality of use of the affected hand in unilateral cerebral palsy
Source: BMC Neurol. 2013 Oct 21;13:152. doi: 10.1186/1471-2377-13-152 (PMC3853931; doi:10.1186/1471-2377-13-152)
Supplement: Additional file 2 — OSAS quality criteria. [file 1471-2377-13-152-S2.docx]

# Additional file 2: OSAS Quality Criteria

## A. Reaching

1. reaching with trunk movement, insufficient shoulder anteflexion and elbow extension

2. reaching with shoulder movement and partial elbow extension, only trunk movement if necessary

3. reaching with shoulder movement and complete elbow extension, only trunk movement if necessary

## B. Grasping

### I. thumb and fingers

1. rummage, rake movement without finger use or pushing in the affected hand by the unaffected hand

2. task oriented active grasping with thumb and finger movements, but not entirely appropriate

3. task oriented active finger opening and closing with thumb opposition

### II. wrist

1. at first contact wrist palmar flexion and ulnar deviation

2. at first contact wrist palmar flexion no ulnar deviation

3. at first contact wrist in neutral position (or dorsal flexion) with ulnar deviation

4. at first contact wrist in neutral position

5. at first contact wrist in dorsal flexion or in appropriate position according to task

## C. Holding

### I. thumb and fingers

1. clenching with arm/hand against trunk or table without finger movement, hand fisted

2. holding with hand no active movement, thumb in or against hand

3. holding with hand no active movement and thumb out of hand

4. holding, varied grip with finger and thumb movement for positioning

### II. wrist

1. continuously wrist palmar flexion and ulnar deviation

2. continuously wrist palmar flexion no ulnar deviation

3. wrist in neutral position (or dorsal flexion) with ulnar deviation

4. wrist in neutral position or wrist extension no ulnar deviation, no variation, not always appropriate

5. wrist extension or neutral position wrist, appropriate for task

## D. Release

1. release from arm

2. passive, pulled out by the unaffected hand or shaking/throwing loose or break up

contact with fisted hand

3. active opening of the hand, gross release, no appropriate object positioning

4. active opening of the hand and appropriate object positioning

E. 1. Unaffected hand used
